# Late-Onset Bloodstream Infection and Perturbed Maturation of the Gastrointestinal Microbiota in Premature Infants

**DOI:** 10.1371/journal.pone.0132923

**Published:** 2015-07-13

**Authors:** Alexander G. Shaw, Kathleen Sim, Paul Randell, Michael J. Cox, Zoë E. McClure, Ming-Shi Li, Hugo Donaldson, Paul R. Langford, William O. C. M. Cookson, Miriam F. Moffatt, J. Simon Kroll

**Affiliations:** 1 Department of Medicine, Section of Paediatrics, Imperial College London, London, United Kingdom; 2 Department of Molecular Genetics and Genomics, National Heart and Lung Institute, Imperial College London, London, United Kingdom; 3 Department of Microbiology, Imperial College NHS Trust, United Kingdom; Vanderbilt University, UNITED STATES

## Abstract

**Background:**

Late-onset bloodstream infection (LO-BSI) is a common complication of prematurity, and lack of timely diagnosis and treatment can have life-threatening consequences. We sought to identify clinical characteristics and microbial signatures in the gastrointestinal microbiota preceding diagnosis of LO-BSI in premature infants.

**Method:**

Daily faecal samples and clinical data were collected over two years from 369 premature neonates (<32 weeks gestation). We analysed samples from 22 neonates who developed LO-BSI and 44 matched control infants. Next-generation sequencing of 16S rRNA gene regions amplified by PCR from total faecal DNA was used to characterise the microbiota of faecal samples preceding diagnosis from infants with LO-BSI and controls. Culture of selected samples was undertaken, and bacterial isolates identified using MALDI-TOF. Antibiograms from bloodstream and faecal isolates were compared to explore strain similarity.

**Results:**

From the week prior to diagnosis, infants with LO-BSI had higher proportions of faecal aerobes/facultative anaerobes compared to controls. Risk factors for LO-BSI were identified by multivariate analysis. Enterobacteriaceal sepsis was associated with antecedent multiple lines, low birth weight and a faecal microbiota with prominent Enterobacteriaceae. Staphylococcal sepsis was associated with Staphylococcus OTU faecal over-abundance, and the number of days prior to diagnosis of mechanical ventilation and of the presence of centrally-placed lines. In 12 cases, the antibiogram of the bloodstream isolate matched that of a component of the faecal microbiota in the sample collected closest to diagnosis.

**Conclusions:**

The gastrointestinal tract is an important reservoir for LO-BSI organisms, pathogens translocating across the epithelial barrier. LO-BSI is associated with an aberrant microbiota, with abundant staphylococci and Enterobacteriaceae and a failure to mature towards predominance of obligate anaerobes.

## Introduction

Late-onset bloodstream infection (LO-BSI) (occurring > 72 hours after birth) is a common complication of prematurity, affecting up to 24.4% of very low birth weight (VLBW) infants (<1,500g).[1] Causative organisms are traditionally grouped by likely mode of acquisition—during insertion of vascular access catheters: *Staphylococcus aureus*, coagulase-negative staphylococci (CoNS), and other Gram-positive skin commensal bacteria;[2–4] and by translocation across the epithelial surface of the immature gastrointestinal (GI) tract: members of the infant gastrointestinal microbiota, in particular Gram-negative enteric bacteria, which account for ca. 20% of all LO-BSI.[5] Mortality is high—ca 25% and 40% for infections caused by Gram-positive and Gram-negative organisms respectively [6]–as is morbidity in survivors: a fourfold increased risk of cerebral palsy and approximately twofold increased risk of neurodevelopmental impairment in the case of Gram-positive LO-BSI.[7]

Host factors predisposing VLBW infants to LO-BSI include an immature and naïve immune system with low levels of passively acquired (maternal) antibody,[8] and fragile skin/mucosal surfaces.[3] These factors, combined with the risks for infection inherent in the delivery of care in the neonatal intensive care unit (NICU) environment: the presence of invasive devices (intravenous (IV) catheters, endotracheal tubes, nasogastric tubes etc.); administration of parenteral nutrition; treatment with multiple courses of broad-spectrum antibiotics; and exposure to antibiotic-resistant organisms;[9] explain why these infants have the highest rates of nosocomial BSIs in the population.[6] Surveillance culture of faeces has been proposed for predicting risk of Gram-negative LO-BSI,[10] but has limited application beyond the search for specific pathogens. Addressing the source of infection is paramount for prevention. Strategies to reduce CoNS and *S*. *aureus* BSIs have centered on good hand hygiene and IV line site care.

We hypothesised that antecedent biomarkers of LO-BSI—clinical features and particular compositions of the developing GI microbiota reflected in the faecal microbiota—may provide an early warning of impending infection and indicate the likely causative organism. To test our hypothesis, we collected detailed daily clinical data and almost every faecal sample passed from birth from >95% of VLBW infants born at our two-site tertiary neonatal unit over a two year period. Each infant who developed LO-BSI was matched to two controls, and the evolving faecal microbiota in samples collected in the period preceding infection characterised on the basis of variation in domains of the 16S rRNA gene revealed by next-generation sequencing.

## Patients and Methods

### Study population

Infants born at <32 completed weeks of gestation admitted to one of the two Imperial College Healthcare NHS Trust NICUs (one at St. Mary’s Hospital, the other at Queen Charlotte’s and Chelsea Hospital) between January 2011 and December 2012 were eligible for inclusion in the study unless considered to be *in extremis* in the first days of life. The NICU is a tertiary center with ~700 admissions per annum. 369 of 388 eligible infants were recruited.

Both hospitals have identical protocols for feeding, prescription of antibiotic and antifungal drugs and the placing of invasive lines (umbilical venous and arterial catheters, and percutaneous intravenous long lines). Staff members work across both sites. Probiotics, H_2_ receptor antagonists, and proton pump inhibitors are not used on the unit. Detailed daily clinical information was collected from the patients’ records.

### Ethics declaration

The study ‘Defining the Intestinal Microbiota in Premature Infants’ (ClinicalTrials.gov Identifier NCT01102738) was approved by West London Research Ethics Committee 2, United Kingdom (Reference number: 10/H0711/39). Parents gave written informed consent for their infant to participate in the study.

### Sample collection

We collected almost every faecal sample produced by each participant from the point of recruitment until discharge. Samples were collected by nursing staff from diapers using a sterile spatula, placed in a sterile DNAase-, RNAase-free Eppendorf tube, stored in a -20°C freezer on the neonatal unit within two hours of collection, and stored at -80°C within five days.

### Case definition, control selection and clinical management

An eligible case for the study was defined as an infant suffering from LO-BSI (organism isolated on/after day three of life), diagnosed using the Vermont Oxford Network criteria,[11] and without preceding or concurrent necrotizing enterocolitis (NEC). Infants with early-onset sepsis were excluded by definition. A diagnosis of BSI attributable to CoNS was based on all of the following three criteria being fulfilled: CoNS isolated from the blood culture, clinical signs of sepsis, IV antibacterial therapy for at least five days after blood culture or until death. Blood cultures were collected under sterile technique and processed using the automated Bactec FX system (BD). Blood isolates were identified by API for the first six months of the study and MALDI-TOF for the remaining eighteen. The diagnosis was made by the attending neonatal consultant and confirmed by an independent neonatologist. Two ‘sequencing control’ infants (no BSI, NEC or culture positive infections diagnosed during admission), of the same postnatal age as each BSI case, were selected based (in order of priority) on gestational age (accounting for prematurity related factors such as gut maturity) and mode of delivery. All such potentially confounding factors were included in the statistical analysis, allowing identification of any that could influence the results. Investigators were not involved in clinical care.

### Bacterial DNA extraction

Faecal samples (200 mg) were processed using the FastDNA SPIN Kit for Soil (MP Biomedicals), incorporating a bead-beating step for mechanical disruption of cells. We have established that this effectively lyses Gram-positive and-negative bacteria in faecal samples (unpublished data). Extractions were performed following the manufacturer’s protocol except that the final elution step was into TRIS (10 mM) low-ethylenediaminetetraacetic acid (EDTA) (0.1 mM) buffer.

### Polymerase Chain Reaction amplification and pyrosequencing of the V3-V5 regions of the bacterial 16S rRNA gene

The V3-V5 region of bacterial 16S rRNA genes was amplified from each DNA sample using a primer pair tagged with individually unique 12-bp error-correcting Golay barcodes.[12–14] Polymerase Chain Reaction (PCR) was performed as previously described.[15] Replicate amplicons were pooled and purified, and pyrosequencing runs were carried out on a 454 Life Sciences GS FLX (Roche) following the Roche Amplicon Lib-L protocol. Replicate samples were spread over all sequencing runs as internal controls and negative controls were included to verify reagents were uncontaminated.

### Bioinformatics

Shotgun processed data were denoised using AmpliconNoise [16] as part of the ‘Quantitative Insights Into Microbial Ecology’ v1.5.0 package [17] followed by chimera-removal with ChimeraSlayer.[18] Sequences were aligned using the SILVA rRNA database (SSU_REF108)[19] for reference and clustered at 97% sequence identity using the UCLUST algorithm [20] into operational taxonomic units (OTUs). Representative sequences were selected and classified using the Ribosomal Database Project Classifier.[21] Rarefaction was performed, removing heterogeneity of sequencing reads per sample.

### Data availability

16S rRNA amplicon data have been deposited at the European Nucleotide Archive under accession number PRJEB6345.

### Bacterial culture and antibiograms

Bacteria were cultured from the frozen faecal sample collected closest before the day of diagnosis of LO-BSI (D_0_) from every case and paired contemporaneous controls (termed ‘culture controls’). CNA agar plates (Oxoid) were used to select for *Staphylococcus* and *Streptococcus* spp. and CHROMagar orientation agar (BD) to differentiate Enterobacteriaceae and *Enterococcus* isolates. All bacterial isolates were identified by matrix assisted laser desorption ionization—time-of-flight (MALDI-TOF) mass spectrometry using a Bruker Microflex LT instrument (Bruker Daltonics). Sadly, blood culture isolates are not routinely retained by the diagnostic laboratory, hence were not available for whole genome sequencing as we would have wished. Further comparison between faecal and blood isolates was therefore made using extended antibiograms. Where there was species concordance between the bacteria isolated from the faecal sample and blood culture, the pure isolates were grown on Iso-Sensitest plates (Oxoid) and appropriate extended antibiograms were performed according to the British Society for Antimicrobial Chemotherapy standardised disc susceptibility protocol.[22] To test the discriminant potential of this typing approach (as previously employed by Köser *et al*.[23]), we examined antibiograms of all CoNS strains isolated from faecal samples collected from babies resident on the NICU at the same time as one index CoNS LO-BSI case.

### Statistical Analysis

Case and control patient characteristics were compared using Student’s t-tests and Chi-squared tests or Fisher’s exact test where appropriate. The conditional logistic regression, generalised linear model and stepwise functions of the R statistical package (version 3.0.2),[24] were used to identify both OTUs that were differentially abundant and clinical and microbial discriminators between LO-BSI and control groups. Factors considered were the relative abundance of each of the top 6 OTUs (comprising 90% of total reads), total proportion of anaerobic organisms, and three measures of microbial diversity (inverse Simpson index, Shannon-Weaver index, Pielou’s evenness). Student’s t-tests (unequal variances) were used to compare OTU count data.

## Results

### Patients

**Fig 1:**
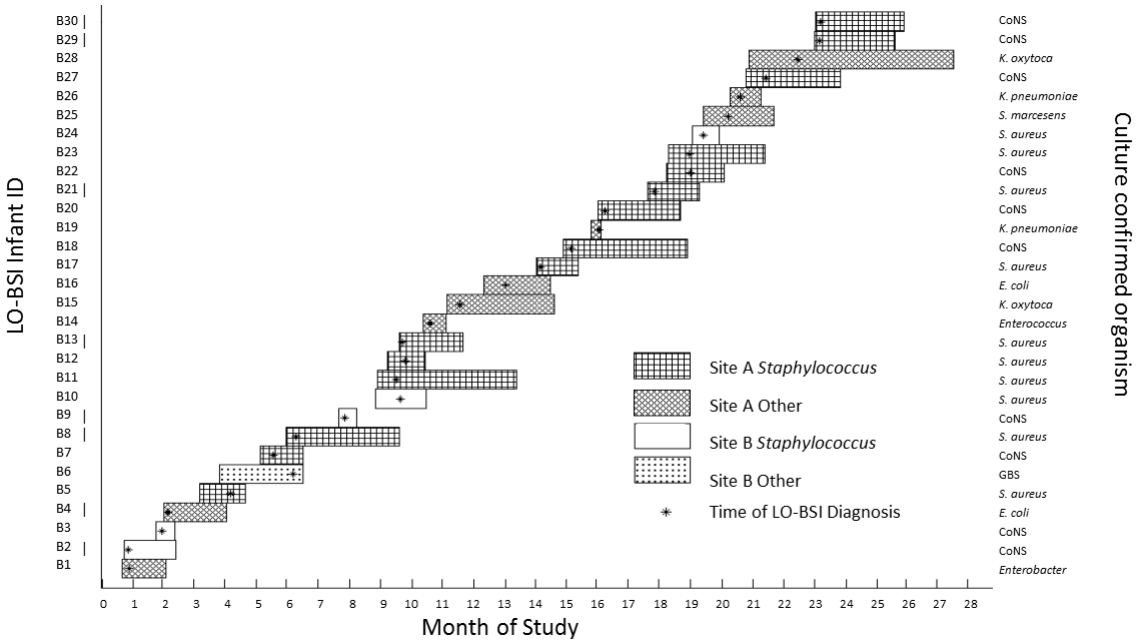
Cumulative cases of LO-BSI over 28 months. Hatching indicates hospital site and whether staphylococcal or other cause. Bar length indicates duration of infant admission; stars indicates date of LO-BSI diagnosis. Marked cases were excluded from the analysis due to lack of samples (n = 6) or lack of sequencing data (n = 2).

369 infants were recruited and 10,928 faecal samples collected. 30 infants were eligible LO-BSI cases during the study period. Fig 1 illustrates the distribution of the cases over the 28 months; infants who could not be included in the analyses are marked by vertical bars (18). The causative LO-BSI organisms are shown in Fig 1. There were no cases of fungal sepsis. Table 1 outlines the cohort clinical characteristics. Prior to diagnosis, compared to controls, infants who developed LO-BSI: were lighter at birth (p = 0.04); required more days of mechanical ventilation (p = 0.007); had more days with invasive lines (p = 0.003); more likely to have multiple lines *in situ* (p = 0.001).

**Table 1:** Summary of the cohort demographics—LO-BSI cases and matched controls.

	Cases (n = 22)	Controls (n = 44)
**Demographics**		
Male (%)	9 (41)	25 (57)
Mean birth weight (SD) (g)	813.0 (257.0) **(p = 0.04)**	959.0 (266.4)
Mean gestation at birth (SD), days	183.6 (14.6)	190.0 (13.2)
Mean postnatal age at D_-1_ (SD), days	18.9 (15.2)	18.9 (15.2)
Admission hospital		
Site A (%)	16 (73)	34 (77)
Site B (%)	6 (27)	10 (23)
Ethnicity		
Black (%)	3 (14)	9 (20.5)
White (%)	8 (36)	23 (52)
Asian (%)	5 (23)	3 (7)
Mixed (%)	6 (27)	9 (20.5)
Mode of delivery		
CS (%)	9 (41)	27 (61)
VD (%)	13 (59)	17 (39)
**Maternal characteristics**		
Maternal infection during pregnancy (%)	3 (14)	5 (11)
Maternal IVAB use at delivery (%)	6 (27)	11 (25)
Maternal PROM (%)	6 (27)	11 (25)
Maternal sepsis/chorioamnionitis (%)	6 (27)	9 (20)
**Intravenous antibiotic use**		
Intravenous antibiotics at given at birth (%)	6 (27)	11 (25)
Mean number of days of IVAB during first week of life (SD), days	2.7 (1.9)	2.3 (1.8)
Mean number of cumulative days of IVAB use prior to D_0_ (SD), days	4.0 (4.5)	2.8 (2.0)
**Invasive lines**		
Mean number of days with an invasive line in situ prior to D_0_ (SD), days	6.3 (4.1) **(p = 0.003)**	3.1 (3.2)
Mean number of total days of each invasive line in situ prior to D_0_ (SD), days	8.1 (5.4) **(p = 0.002)**	3.5 (3.9)
Presence of invasive line/s prior to D_0_ (%)	17 (77) **(p = 0.01)**	23 (52)
Presence of multiple invasive lines prior to D_0_ (%)	12 (55) **(p = 0.001)**	9 (20.5)
**Respiratory support requirement**		
Mean number of days requiring ventilation support (HFOV or conventional ventilation) prior to D_0_ (SD), days	7.2 (8.6) **(p = 0.007)**	1.5 (2.4)
Mean number of days requiring CPAP (no oxygen), prior to D_0_ (SD), days	4.3 (6.3)	7.1 (8.1)
Mean number of days requiring CPAP with supplemental oxygen, prior to D_0_ (SD), days	4.9 (3.8)	5.0 (6.9)
**Metadata related to sample closest to D_0_**		
Mean number of postnatal days sample closest to D_0_ analysed, (SD)	17.5 (15.3)	18.0 (16.0)
Mean weight closest to D_0_, (SD) (g)	899.5 (459.2)	1052.7 (431.7)

Abbreviations—SD, standard deviation; D_-1_, day prior to diagnosis, D_0_, day of LO-BSI diagnosis or postnatal age of matched control; CS, Caesarean-section; VD, vaginal delivery; IVAB, intravenous antibiotics; PROM, prolonged rupture of membranes; HFOV, high frequency oscillation ventilation; CPAP, continuous positive airways pressure. *P* values of significant differences between LO-BSI cases and controls are denoted in bold

### 454 Pyrosequencing and initial data processing

An average of 2 samples per subject-week were analysed from the 44 ‘sequencing control’ infants to characterise the maturation of the normal GI microbiota. From each LO-BSI infant weekly faecal samples from birth and a median of five faecal samples collected in the two weeks prior to D_0_ were characterised. There were 434 and 153 samples successfully processed from sequencing controls and LO-BSI infants respectively. After denoising and chimera removal 2,635,031 reads remained and were de-multiplexed. The mean number of reads was 4,489 per sample. Singletons (sequences present only once in a sample) and OTUs present in only one sample were removed. For maximum data retention, sample reads were rarefied to 800, with rarefaction curves showing this to be sufficient to capture the OTU diversity as measured by the Shannon diversity index (S1 Fig).

### Bacterial communities in control samples

The most abundant OTUs (90% of reads) were Klebsiella, Staphylococcus, Enterococcus, Escherichia, Clostridium and Bifidobacterium (data derived from one faecal sample per week per sequencing control) (S2 Fig; for full data see S3 Fig). Bacterial communities evolve rapidly with multiple OTUs having significantly varying differential abundance with postnatal age (p<0.05) and by delivery method (p<0.05).

### Anaerobic succession of the GI microbiota

**Fig 2:**
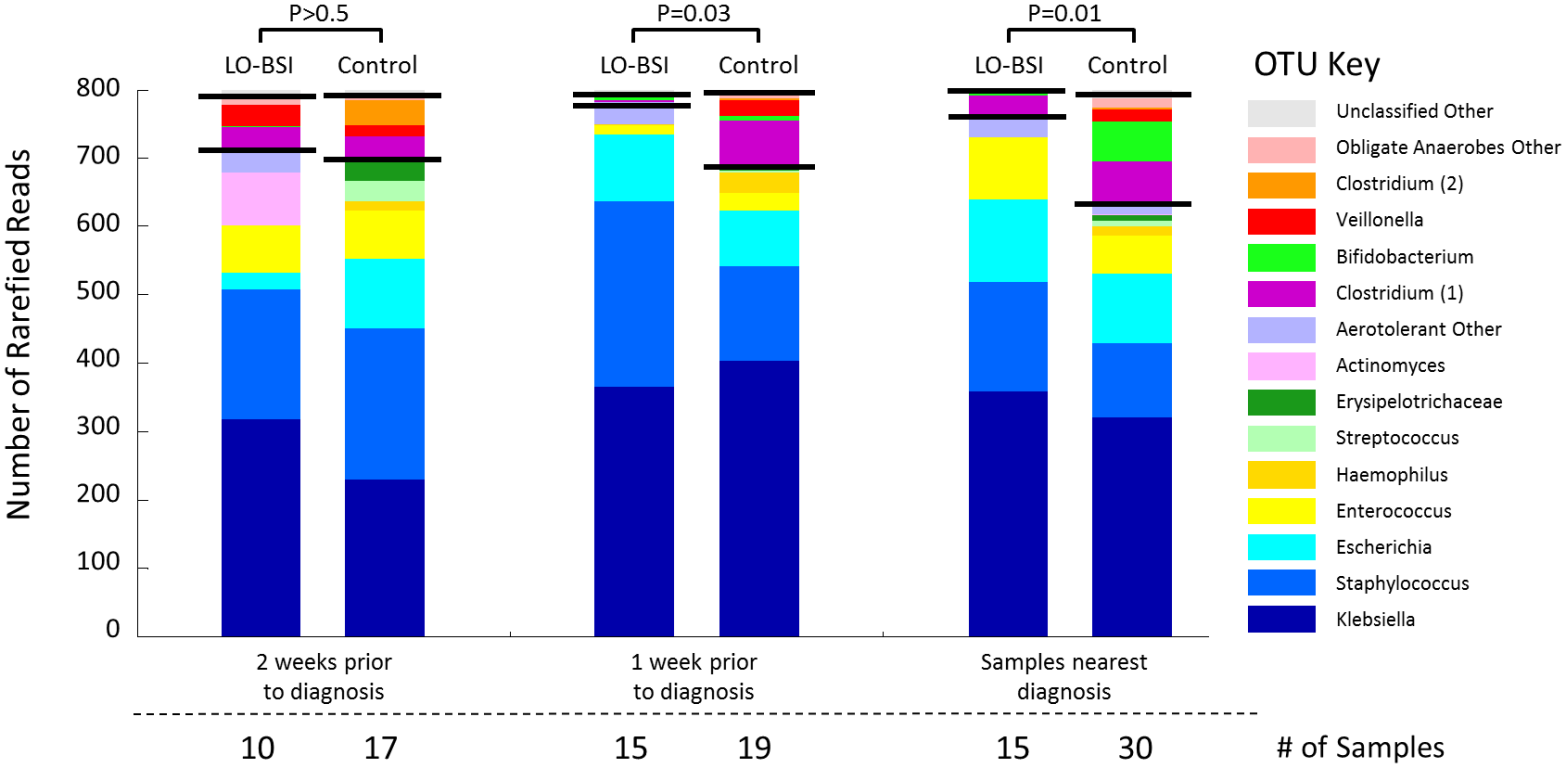
Bacterial faecal community structure developing prior to D_0_. Data generated using one sample weekly from LO-BSI infants and controls. Black lines on the stacked bars divide the identified OTUs: lower segment comprises aerobes/facultative anaerobes, upper segment obligate anaerobes.

OTUs were categorised as aerobes/facultative anaerobes (“aerotolerant”) and obligate anaerobes, based on NCBI annotations.[25] During the first week of life, there was no difference in mean OTU reads between these two categories in infants who developed LO-BSI (n = 13) and controls (n = 26). In older infants developing LO-BSI, and their controls, there was a significantly higher proportion of obligate anaerobes one week prior to LO-BSI diagnosis in faecal samples from the control group than from cases (p = 0.03) and this divergence was most extreme in the samples collected closest to D_0_ (p = 0.01) (Fig 2).

**Fig 3:**
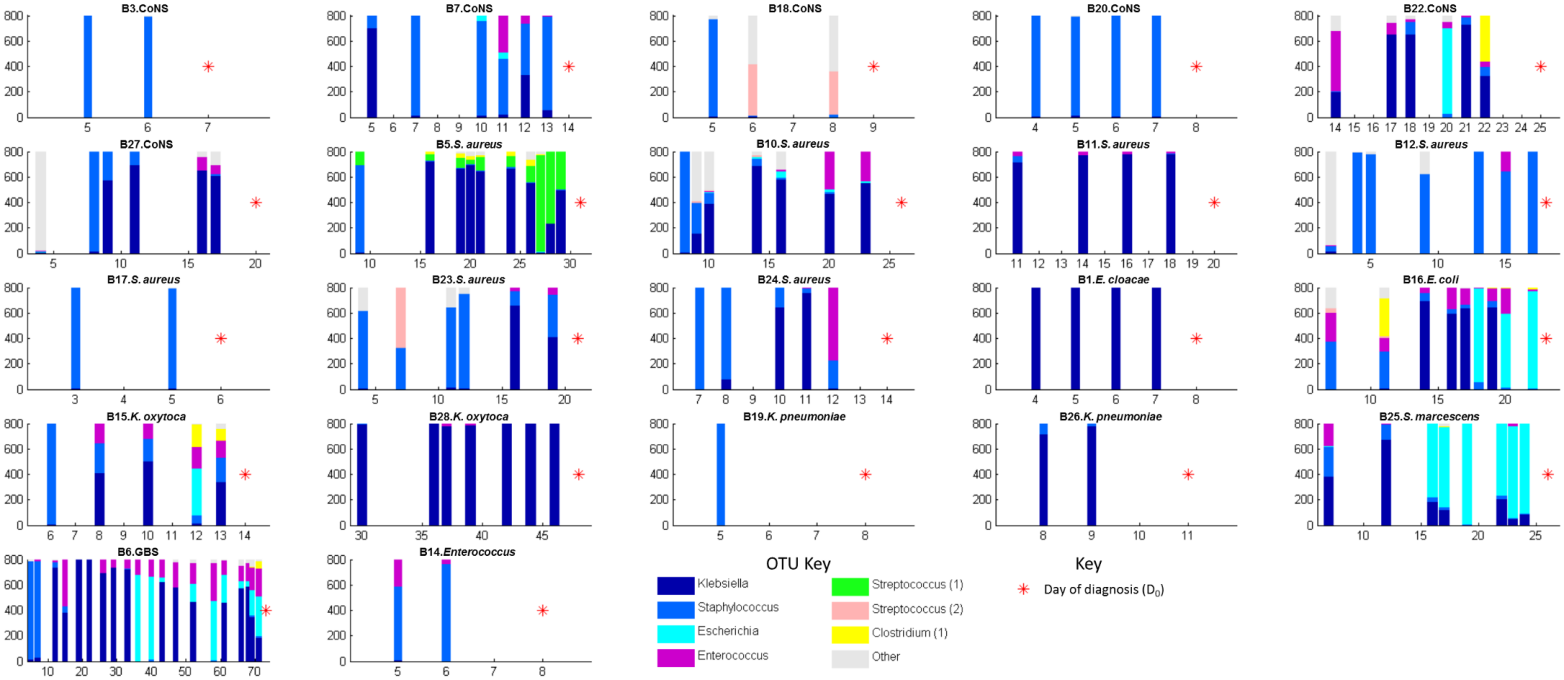
Development of the faecal microbiota in infants with LO-BSI. Bar charts are identified by the infant ID and the microbe cultured from the blood. Stacked bars show composition of faecal microbiota on days indicated (x-axis). Red stars indicate the day of life of LO-BSI diagnosis.

The most abundant OTUs (90% of reads) in faecal samples collected closest to D_0_ in LO-BSI cases were Klebsiella, Staphylococcus, Escherichia, Enterococcus and Clostridium (see S4 Fig **)**. Logistic regression indicated a significant association (p = 0.047) between abundant Staphylococcus OTU reads in the first week of life (mean reads = 563 in cases (n = 13), 337 in controls (n = 26)) and later LO-BSI. The development of the GI microbiota for each infant with LO-BSI is shown in Fig 3.

### Logistic regression

Anticipating differences in risk factors for LO-BSI caused by different bacteria, logistic regression was used in a first round of analysis to identify clinical factors (Table 1) and microbial population signals that discriminated Enterobacteriaceae and “Other” (*Enterococcus*, GBS) LO-BSI cases together from both Staphylococcal cases and Controls. Conditional univariate analysis highlighted the presence of multiple lines (p = 0.006), lower weight at birth (p = 0.02), lower gestational age at birth (p = 0.008), more days of mechanical ventilation (p = 0.03), and more cumulative total number of days of each invasive line present (‘line days’) (p = 0.02), as all being associated with Enterobacteriaceae and “Other” LO-BSI. These factors, together with those significant at the 0.05 < p < 0.1 level (‘trending factors’: number of Escherichia OTU reads and cumulative days on antibiotics), were included in a multivariate analysis, using a stepwise algorithm to retain key variables. Three factors were retained in the final model: the presence of multiple lines, weight at birth, and number of Escherichia OTU reads.

Infants not identified in this first round of analysis were included in a second round, aiming to discriminate staphylococcal LO-BSI cases from the rest. Conditional univariate analysis highlighted more days of mechanical ventilation (p = 0.03), more days with any lines (p = 0.01) and more cumulative ‘line days’ (p = 0.01). Multivariate analysis including trending factors (number of Staphylococcus OTU reads) gave a final model retaining: days of mechanical ventilation, days with any lines and number of Staphylococcus OTU reads.

### LO-BSI screening criteria

**Fig 4:**
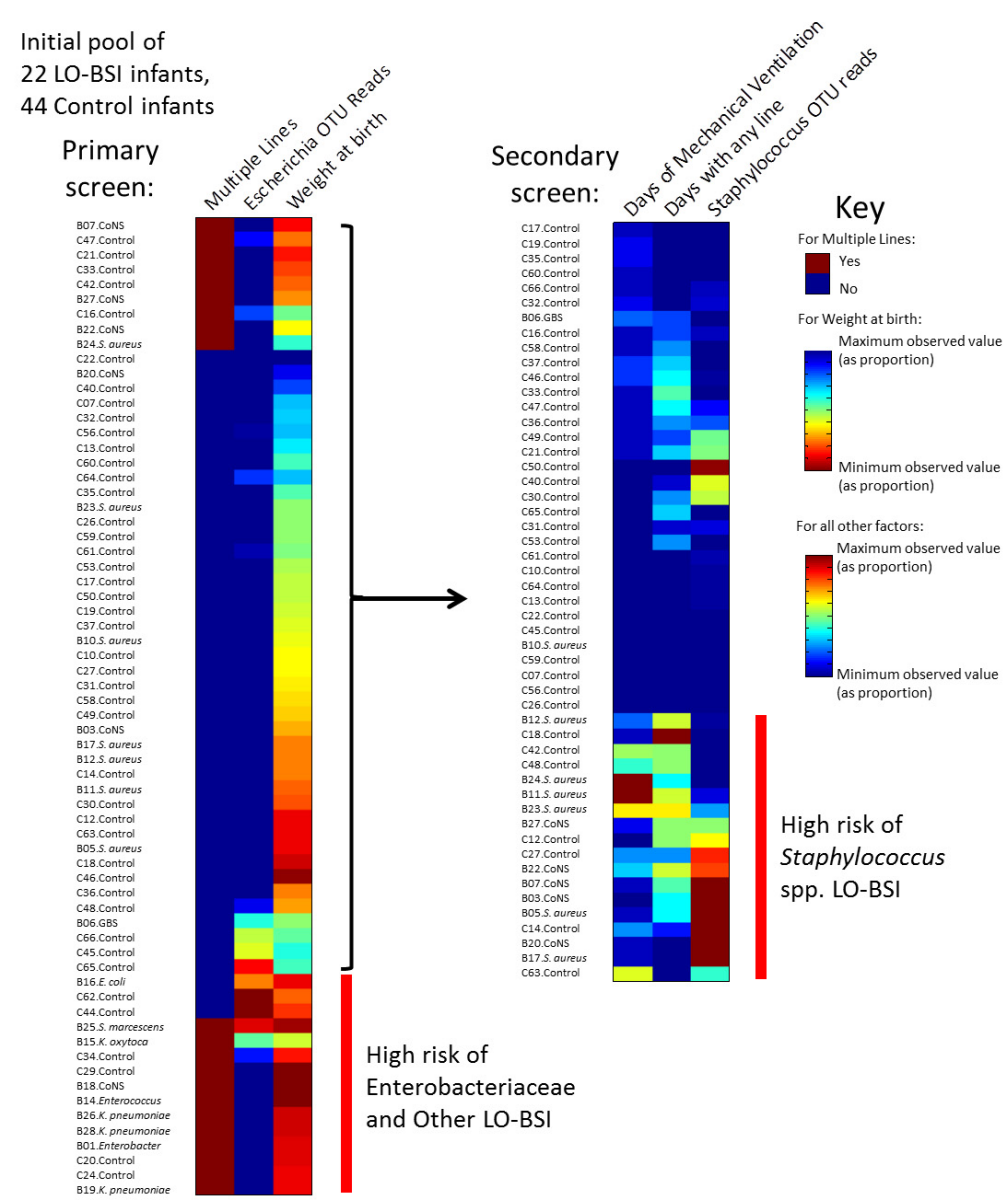
Clustering of LO-BSI cases and controls into risk groups. Three heat maps display clinical information on the day prior to diagnosis, and sequencing information from the faecal sample closest to D_0_. A distinct cluster of infants at high risk of Enterobacteriaceae and “Other” LO-BSI is indicated. Infants not within this category undergo a second round of screening, identifying infants at high risk of Staphylococcal LO-BSI.

Using the risk factors identified by multivariate analysis, hierarchical clustering performed on the dataset correctly classified 20/22 cases and 31/44 control infants (Fig 4).

The potential utility of this approach in defining risk depends crucially on inclusion of microbiota data. A repeat of the two-stage analysis after its removal led to greatly lowered sensitivity and specificity. If alternatively all LO-BSI cases are combined and compared to controls, a multivariate model identifies risk associated with increased days of mechanical ventilation and more cumulative ‘line days’ with fewer false positives (correct classification of 40/44 controls) but a reduced sensitivity (only 15 cases identified).

### Culture and Antibiogram data

**Fig 5:**
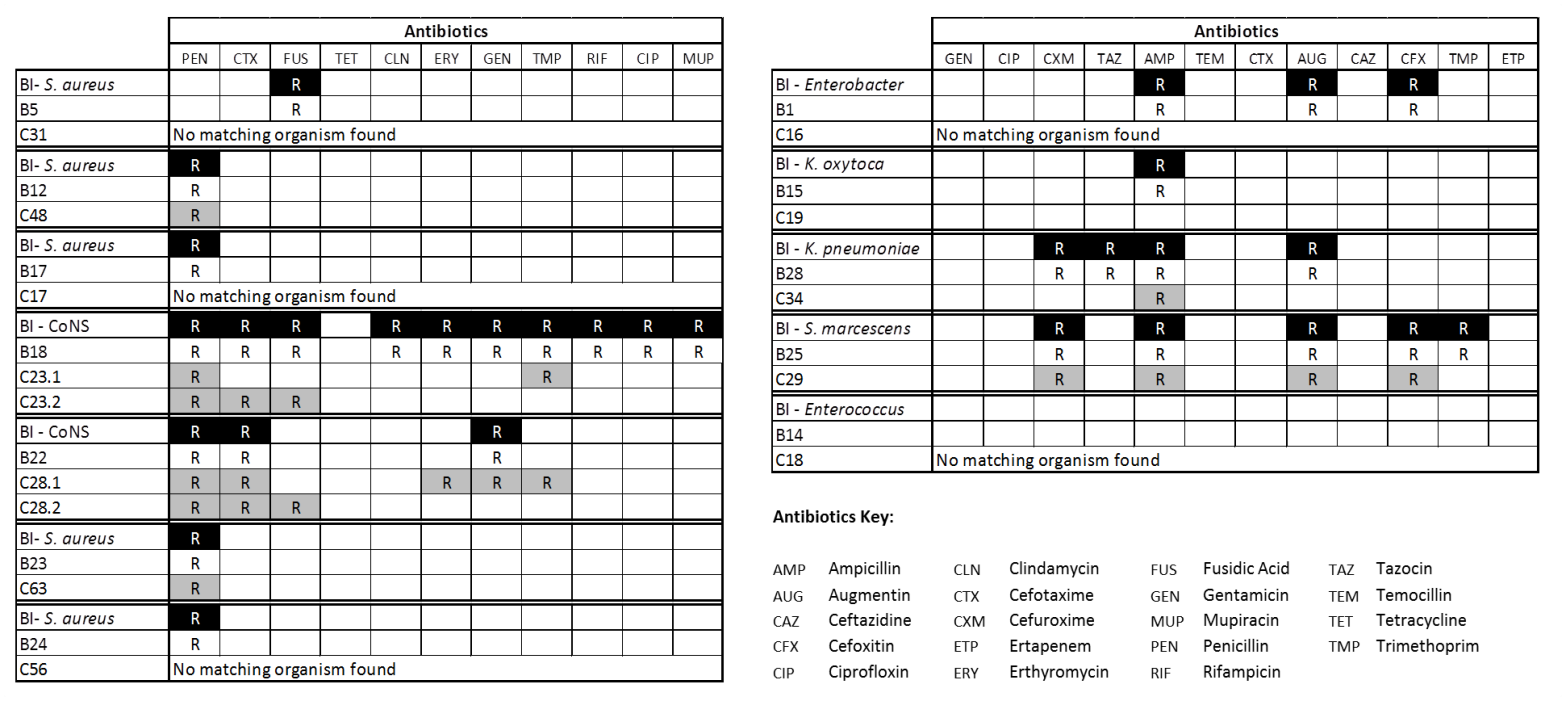
Antibiogram concordance of isolates found in faecal samples of LO-BSI infants and contemporaneous controls. Table aligns antibiograms of blood isolate (BI), corresponding faecal isolate (B) and isolates from the contemporaneous control (C). R indicates antibiotic resistance. In two cases faecal samples from controls contained two different staphylococcal strains: both antibiograms are shown.

Antibiotic resistance profiles (extended antibiograms) had been determined for the organisms isolated from the bloodstream of 21 LO-BSI infants (information on the isolate from infant B16 was not available). Faecal samples collected closest to D_0_ from these infants yielded microbes with antibiograms that matched the bloodstream isolate in 12 cases (Fig 5), compared to only 3 matches to the index case bloodstream isolate in microbes isolated from culture control infants (p = 0.01). In consideration of the possibility that matches between bloodstream and faecal isolates might be a chance finding, the antibiogram of the bloodstream isolate from one CoNS LO-BSI case (B18) was compared to those of microbes isolated by selective culture for CoNS from faecal samples collected from all eight contemporaneous residents on the same NICU site. Fourteen morphologically distinct isolates were recovered from these babies, and extended antibiograms were established for each. Eleven different profiles were observed, but none matched the LO-BSI organism isolated from B18. We infer that antibiograms matching between faecal and bloodstream isolates in a septic infant may reflect bacterial translocation between GI tract and the circulation.

## Discussion

We have found that the occurrence of LO-BSI in premature infants is correlated with an aberrant development of the GI microbiota as reflected in faecal composition—a delay, or a reversal, in the shift to anaerobiosis. In newborn infants the initial GI microbiota is aerotolerant, but becomes increasingly dominated by anaerobes over the first weeks/months of life,[26] a process that appears to occur more gradually in healthy premature infants.[27–30] In our population of premature infants who developed LO-BSI, Staphylococcus and/or Gram-negative enteric OTUs dominate the GI microbiota right up to the time of the diagnosis of sepsis, as long as several weeks after birth. Staphylococcal prominence in the faecal microbiota has been noted in neonatal sepsis in one other small 16S gene sequencing study,[31] while Mai *et al*., [32] using the same approach, reported a bloom of Gram-negative enteric organisms shortly before the diagnosis of LO-BSI in premature infants.

What might the mechanism be? *S*. *aureus* has been shown to perturb the normal microbiota of the human colon, reducing the proportion of anaerobic species such as *Bifidobacterium* and *Lactobacillus*,[33] often employed in probiotic formulations. This may explain the disappointing lack of efficacy of trials of probiotics in the prevention of LO-BSI.[34 The immaturity of the infant, with potential oxygenation of the GI tract through a leaky epithelium, and the NICU setting itself (antibiotic treatments and oxygen-rich ventilation) may also contribute to this lack of anaerobic progression.

It has had been demonstrated that some obligate anaerobes prevent bacterial translocation by increasing epithelial integrity.[35, 36] Additionally, the absence of anaerobic bacteria increases the risk that facultative anaerobes may translocate across the intestinal barrier [37]–either the predominant staphylococci/Enterobacteriaceae present, or other components of the microbiota. In our study twelve infants’ infections fell into the first of these categories—seven suffering from staphylococcal bloodstream infection (five *S*. *aureus*, two CoNS), and five with Gram-negative sepsis. The organism from the bloodstream was indistinguishable from a dominant component of their faecal microbiota. In the second category, one infant with enterococcal LO-BSI had the same enterococcus as a minor component of his staphylococcal-dominated faecal microbiota. In the remaining nine infants the source of the bloodstream infection was not clear. Possibilities to consider included the GI microbiota, the skin (breached by invasive lines) and the intubated airway. In three cases where the bacterial species responsible for bloodstream infection could not be detected in the faecal microbiota, there were clear risk factors for infection elsewhere—invasive lines and mechanical ventilation. In two further cases (both caused by CoNS) where there were no such risk factors, the faecal microbiota were dominated by a Staphylococcus OTU. It may be that the mismatch of antibiograms between the bloodstream isolate and the faecal isolate examined simply reflected a failure to capture a relatively minor component of the faecal microbiota. The collection of swabs and aspirates to characterize the epidermal and pulmonary microbiota, in addition to swabs of line insertion sites and indwelling devices, could further clarify potential sources of infection. This wealth of information could also allow the investigation of interactions between the microbiota at both environmental interfaces and different body sites.

Carl *et al*.[38] demonstrated an origin in the GI microbiota for organisms responsible for 7/11 cases of LO-BSI in a VLBW infant cohort, but did not consider staphylococcal bloodstream infection relevant, regarding these as most likely originating from the skin. While CoNS are indeed common skin colonists,[2, 4] detailed typing of strains collected from multiple sites in individual patients has demonstrated that blood isolates often match mucosal rather than skin strains.[39] Indeed, *S*. *epidermidis* (a coagulase negative *Staphylococcus*) has been shown to translocate from the gut with the same efficiency as *E*. *coli* and more readily than *K*. *pneumoniae* in a mouse model.[40] The seven infants in our study with staphylococcal LO-BSI, where the causative strain was also identified in the faecal microbiota, attest to the fact that staphylococcal LO-BSI may commonly arise by translocation of organisms from a GI microbiota habitat.

LO-BSI is an important cause of mortality and morbidity in preterm infants, and our study implicates the GI microbiota as an important reservoir of infection, not only for Gram-negative organisms, but also for staphylococci. This focuses attention on strategies for the timely detection of GI dysbiosis. Should they be translated to a diagnostic test, our results suggest that it should be possible to identify babies who are destined develop LO-BSI with a sensitivity of 91% and specificity of 70%, out-performing single CRP measurements in the prediction of late onset sepsis.[41] Our analysis identified significant features both in the composition of the microbiota and a failure to mature—persistent staphylococci and Enterobacteriaceae and paucity of obligate anaerobes—which, whilst not necessarily causal, are correlated with a progression to LO-BSI. At present, conducting complete microbiota analysis in a manner timely enough to impact infant management is not practical. This study however points the way towards development of specific surveillance tools, such as organism-targeted qPCR, to monitor the maturation of the GI microbiota, perhaps combined with strategies to alter the microbiota to a “safer” composition. This could be achieved through modulation of the gut microbiota by treatments shown to reduce LO-BSI (such as lactoferrin),[42] the judicious use of probiotics accompanied by a selective decontamination protocol, or by more radical bacteriotherapy.

## Supporting Information

S1 Fig Rarefaction curves for sequencing data. A randomised selection of 10% of the rarefaction curves for the dataset. Black dashed line shows the chosen cut off value for rarefaction.
(TIF)

S2 Fig Bacterial fecal community structure in control infants by postnatal week and mode of delivery. Data generated using one sample per week from sequencing control infants. When the same descriptive label (genus, family) is attached to multiple OTUs, these are numbered sequentially—no OTUs are combined.
(TIF)

S3 Fig The GI microbiota of 44 control infants. Samples are categorised along the x axis, grouped by infant and then chronologically with the earliest sample on the left. Infants are grouped by admission hospital. Colour intensity indicates the number of rarefied reads from each OTU that are found in a sample, as shown by the coloured bar.
(TIF)

S4 Fig Heat map of bacterial communities in fecal samples collected closest to D_0_ from LO-BSI infants and their matched controls. LO-BSI infant samples are grouped by LO-BSI organisms and sequencing control samples according to LO-BSI organism of their matched case. Color intensity indicates number of rarefied reads from each OTU in a sample.
(TIF)
