# Supplementary figures and images for: Late-Onset Bloodstream Infection and Perturbed Maturation of the Gastrointestinal Microbiota in Premature Infants
Source: PLoS One. 2015 Jul 13;10(7):e0132923. doi: 10.1371/journal.pone.0132923 (PMC4500406; doi:10.1371/journal.pone.0132923)

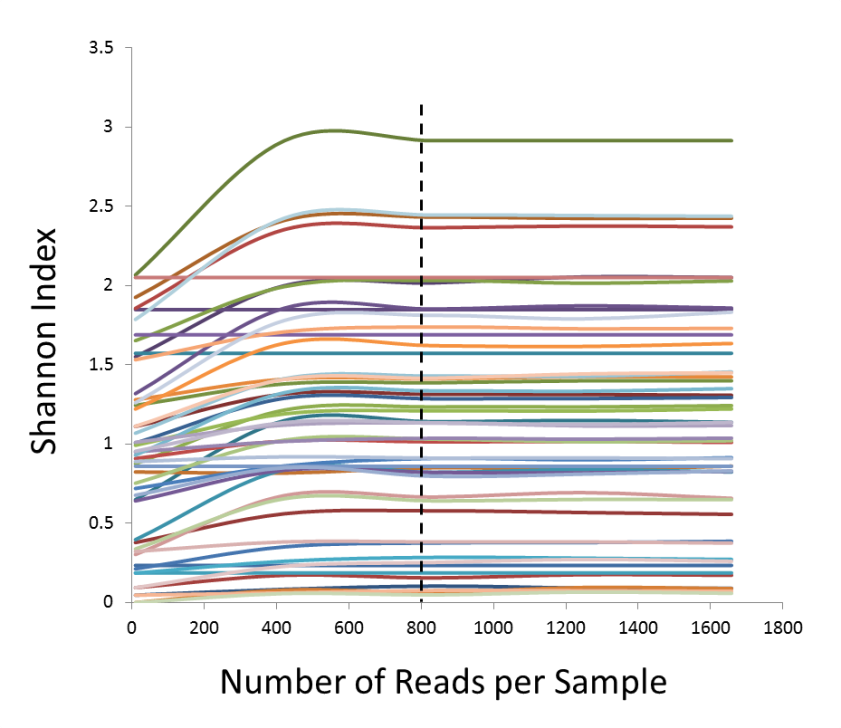

Supplement: S1 Fig — A randomised selection of 10% of the rarefaction curves for the dataset. Black dashed line shows the chosen cut off value for rarefaction. (TIF) [file pone.0132923.s001.tif]

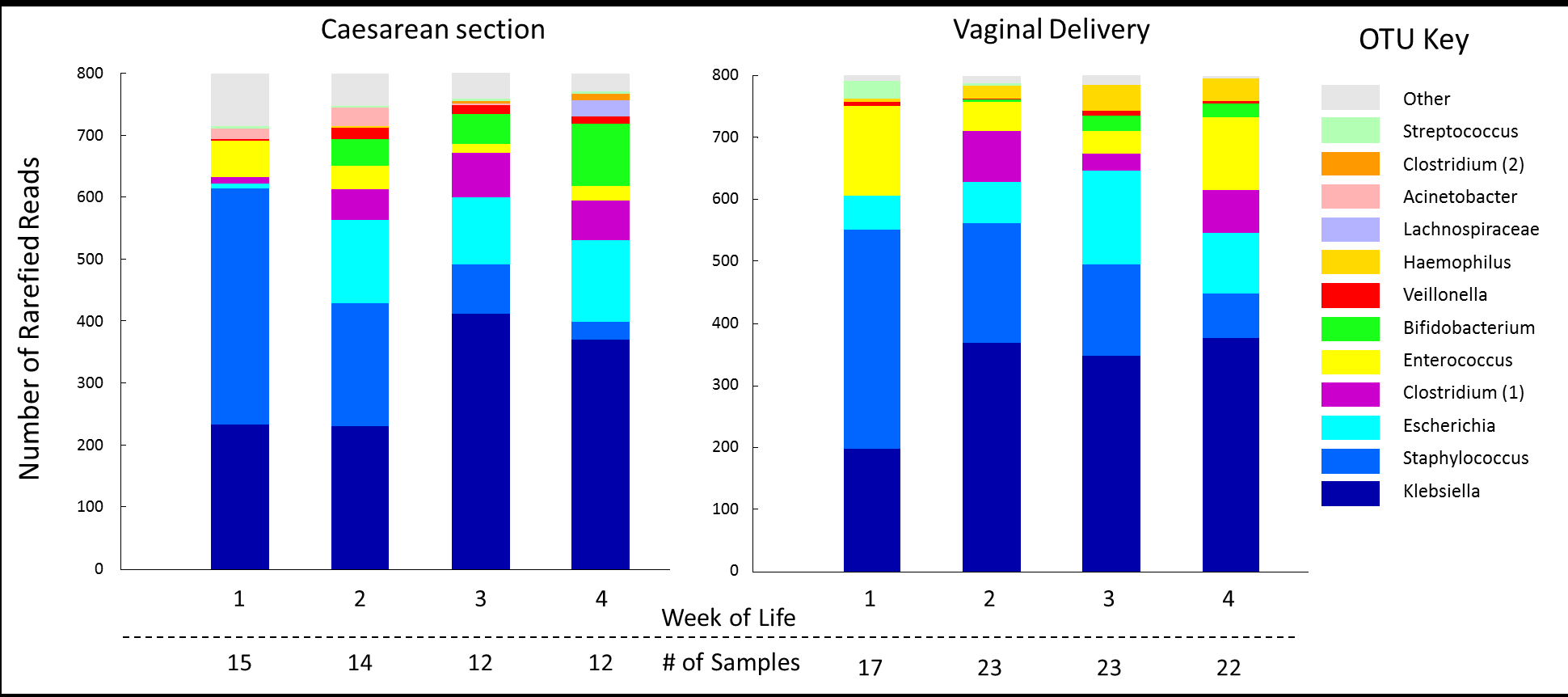

Supplement: S2 Fig — Data generated using one sample per week from sequencing control infants. When the same descriptive label (genus, family) is attached to multiple OTUs, these are numbered sequentially—no OTUs are combined. (TIF) [file pone.0132923.s002.tif]

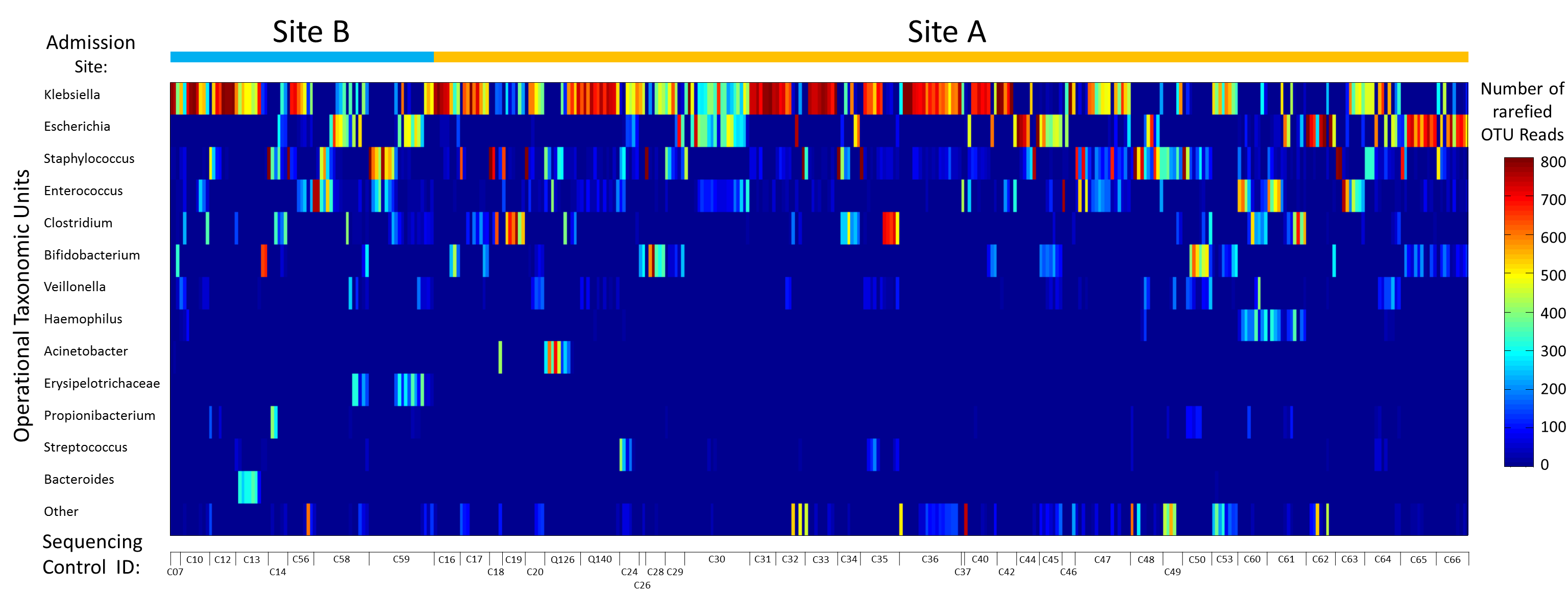

Supplement: S3 Fig — Samples are categorised along the x axis, grouped by infant and then chronologically with the earliest sample on the left. Infants are grouped by admission hospital. Colour intensity indicates the number of rarefied reads from each OTU that are found in a sample, as shown by the coloured bar. (TIF) [file pone.0132923.s003.tif]

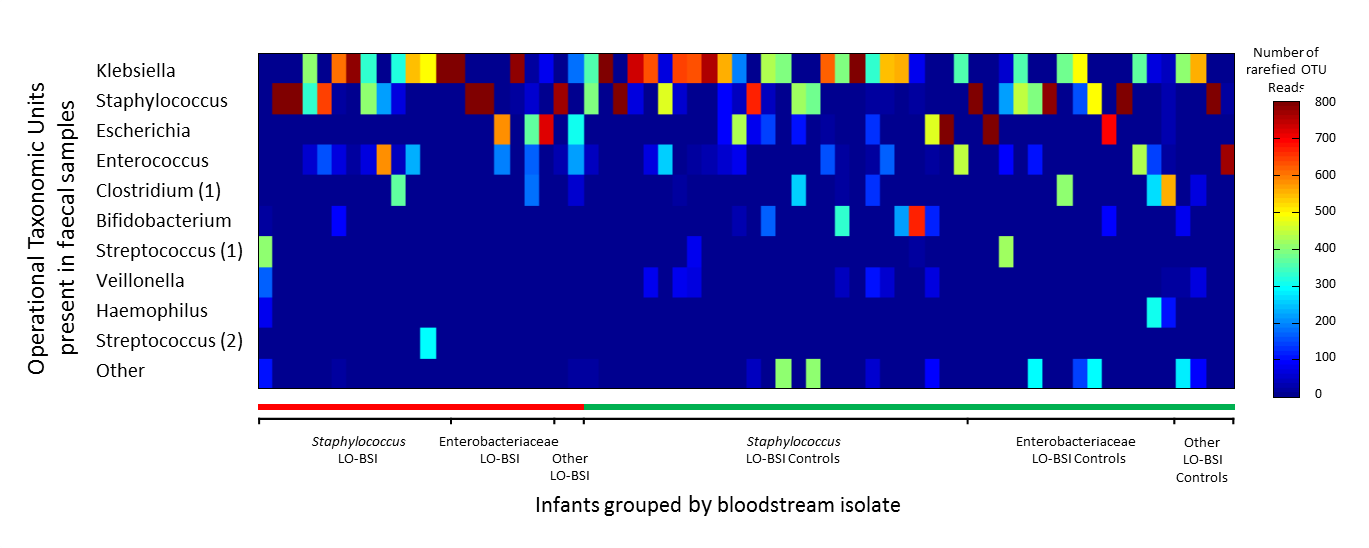

Supplement: S4 Fig — LO-BSI infant samples are grouped by LO-BSI organisms and sequencing control samples according to LO-BSI organism of their matched case. Color intensity indicates number of rarefied reads from each OTU in a sample. (TIF) [file pone.0132923.s004.tif]
